# Effects of different straw biochar combined with microbial inoculants on soil environment in pot experiment

**DOI:** 10.1038/s41598-021-94209-1

**Published:** 2021-07-19

**Authors:** Yuqi Qi, Haolang Liu, Jihong Wang, Yingping Wang

**Affiliations:** 1 College of Resource and Environment, Jilin Agricultural University, Changchun, Jilin China; 2 College of Traditional Chinese Medicinal Materials, Jilin Agricultural University, Changchun, Jilin China

**Keywords:** Ecology, Environmental sciences

## Abstract

Ginseng is an important cash crop. The long-term continuous cropping of ginseng causes the imbalance of soil environment and the exacerbation of soil-borne diseases, which affects the healthy development of ginseng industry. In this study, ginseng continuous cropping soil was treated with microbial inocula using broad-spectrum biocontrol microbial strain *Frankia* F1. Wheat straw, rice straw and corn straw were the best carrier materials for microbial inoculum. After treatment with microbial inoculum prepared with corn straw biochar, the soil pH value, organic matter, total nitrogen, available nitrogen, available phosphorus, and available potassium were increased by 11.18%, 55.43%, 33.07%, 26.70%, 16.40%, and 9.10%, the activities of soil urease, catalase and sucrase increased by 52.73%, 16.80% and 43.80%, respectively. A Metagenomics showed that after the application of microbial inoculum prepared with corn straw biochar, soil microbial OTUs, Chao1 index, Shannon index, and Simpson index increased by 19.86%, 16.05%, 28.83%, and 3.16%, respectively. Three classes (*Alphaproteobacteria, Gammaproteobacteria and Sphingobacteria*) were the dominant bacteria in ginseng soil, and their abundance increased by 7.87%, 9.81% and 1.24%, respectively, after treatment with microbial inoculum with corn straw biochar. Results indicated that the most effective treatment in ginseng soil would be the combined application of corn straw biochar and *Frankia* F1.

## Introduction

Ginseng (*Panax ginseng* Meyer) is a valuable medicinal plant. Its root is used to enhance organ function and prevent various disorders^1–4^. However, long-term, continuous, crop rotation has created favorable conditions for the reproduction and spread of pathogenic fungi that affect ginseng production. This practice has allowed diseases to become increasingly prominent, threatening crop yield and quality^5–7^. At present, biological control has been reported in many crops^8,9^_._ Biocontrol practices are the current focus of disease control research because they are environmentally stable, nontoxic, and efficient. Therefore, the application of biological pesticides and the development of microbial fungicides are the inevitable trend in plant disease control. Studies in China and other countries have shown that various antagonistic antifungal agents such as *Chaetomium globosum*^10^ and *Bacillus amyloliquefaciens* can inhibit ginseng pathogens^11^.

Biochar is a kind of stable and highly aromatic solid material produced by pyrolysis and carbonization of biomass at high temperature under oxygen limitation. It has a unique structural characteristics and has attracted wide attention. Its physical structure and chemical properties have good benefits to soil, and it is a potential soil conditioner and adsorbent^12,13^. Not only can biochar reduce nutrient leaching^14,15^ and improve soil structure, it can also provide nutrients and living niches for soil microorganisms^16^, reduce the competition among microbes, protect the beneficial soil microorganisms^17^, improve the soil microbial community structure, and enhance soil bacterial diversity^18–20^. Biochar may be an effective way to improve the quality of acid soils^21^. For example, Mao et al.^22^ pointed out that adding bamboo charcoal and commercial microbial inoculum to pig manure composting caused changes in the relative abundance of *Firmicutes* and *Proteobacteria* in the bacterial community during the high-temperature period of composting, thus reducing the production of CH_4_, N_2_O and NH_3_ by 69%-80%, 45% and 19%-29%, respectively. Duan et al.^23^ found that, compared with the control treatment, the addition of wheat straw charcoal and microbial inocula (extracted from fresh cow dung) into cow dung compost significantly increased the number and abundance of microorganisms in the compost.

*Frankia* is a type of actinomycetes that can form nodules on the roots of non-leguminous plants and perform symbiotic nitrogen fixation^24^. Plants that can form nodule with *Frankia* symbiosis are collectively called actinorhizal plants. They have strong symbiotic nitrogen fixation ability and are widely distributed. They are important nitrogen suppliers in terrestrial ecosystems, so they have a wide application prospect in agriculture and forestry. Thus, *Frankia* has the potential to be used in nature as a powerful resource^25^. Other studies have found that *Frankia*, like other actinomycetes, can produce antimicrobial substances that inhibit the growth of microorganisms, such as *Escherichia coli*, *Staphylococcus aureus*, *Candida albicans*, and *Penicillium chrysogenum*^26^. Lihua^27^ showed that *Frankia* could improve the nutrient composition, organic matter, total nitrogen, available potassium and available nitrogen contents in *Casuarina casuarina* forest land, with the increase in available nitrogen being the largest.The biocontrol effect of actinomycetes on plant diseases depends on the stability of their colonization in the environment, which is affected by many factors, such as temperature, humidity, soil type, pH, nutritional status, and microbial community diversity of the soil^28^.

Recently, new generation metagenomics has been applied to comprehensively analyze the structure of the soil microbial community based on updated high-throughput sequencing technology^29^. This method could rapidly provide accurate high-volume sequence data and offer an opportunity to achieve a high throughput and deeper insight into the effects of different treatments on the composition of microbial communities^30^. We set up four treatments, i.e. CK (control treatment: no fertilizer and biochar), A (wheat straw biochar with microbial inoculum), B (rice straw biochar with microbial inoculum), and C (corn straw biochar with microbial inoculum), to analyze soil physical and chemical properties, fertility, enzyme activity and bacterial richness, evenness, community composition, and structural changes. Extensive studies to identify effective microbial inoculum can accelerate soil ecological recovery, and shorten the interval years during continuous cropping of ginseng; these advances could have far-reaching significance and potential applications in agriculture.

## Results

### Wide-spectrum verification of *Frankia* F1 against pathogenic fungi of ginseng

**Figure 1:**
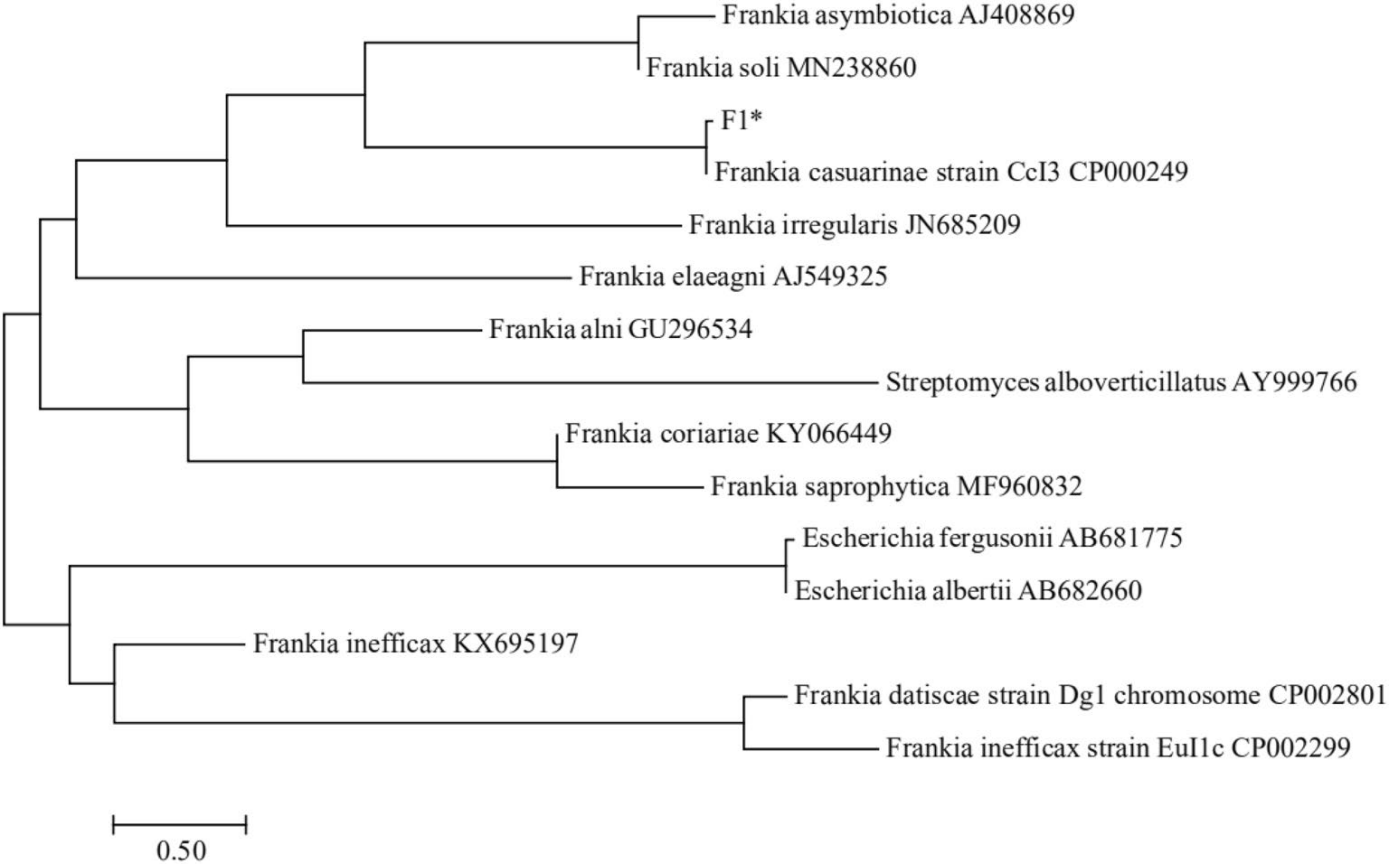
Phylogenetic tree constructed by *Frankia* F1 based on 16S rRNA gene sequence. Using software MEGA7.0 (https://www.megasoftware.net/home) to construct.

The phylogenetic tree of the F1 strain is shown in Fig. 1. It has 100% homology with the *Frankia casuarinae* strain CCl3, which has been published in GenBank. The sequence accession number is GU296535. The fungistasis spectrum test of *Frankia* F1 against pathogenic fungi of ginseng (Table 1) showed that *Frankia* F1 had significant inhibitory effects on *Fusarium solani*, *Sclerotinia schinseng*, *Cylindrocarpon destructans*, *Alternaria panax*, and *Rhizoctonia solani* with inhibition rates of 80.23%, 73.91%, 72.12%, 70.87%, and 68.31%, respectively. It also showed some inhibitory effect on *Phytophthora cactorum* and *Botrytis cinerea*. In conclusion, *Frankia* F1 has a broad-spectrum fungistatic effect against ginseng pathogenic fungi under the conditions of the in vitro plate test.

**Table 1:** Inhibition of activity of *Frankia* F1 against pathogenic fungi of ginseng.

Pathogenic fungus	Colony diameter(mm)	Fungal inhibition rate(%)
*Fusarium solani*	22.45 ± 3.12 g	80.23 a
*Cylindrocarpon destructans*	23.13 ± 4.06 f.	72.12 c
*Phytophthora cactorum*	35.32 ± 3.63 a	63.82 g
*Alternaria panax*	29.58 ± 2.98 d	70.87 d
*Rhizoctonia solani*	30.37 ± 2.37 c	68.31 e
*Sclerotinia schinseng*	25.28 ± 5.10 e	73.91 b
*Botrytis cinerea*	31.18 ± 3.27 b	66.37 f.

All the presented values are means of three replicates. Means were subjected to analysis of variance and were separated by the LSD test. Letters represent the significant differences among the mean values and the “ ± ” is followed by the standard error values of the means.

### Preparation of microbial inocula

**Figure 2:**
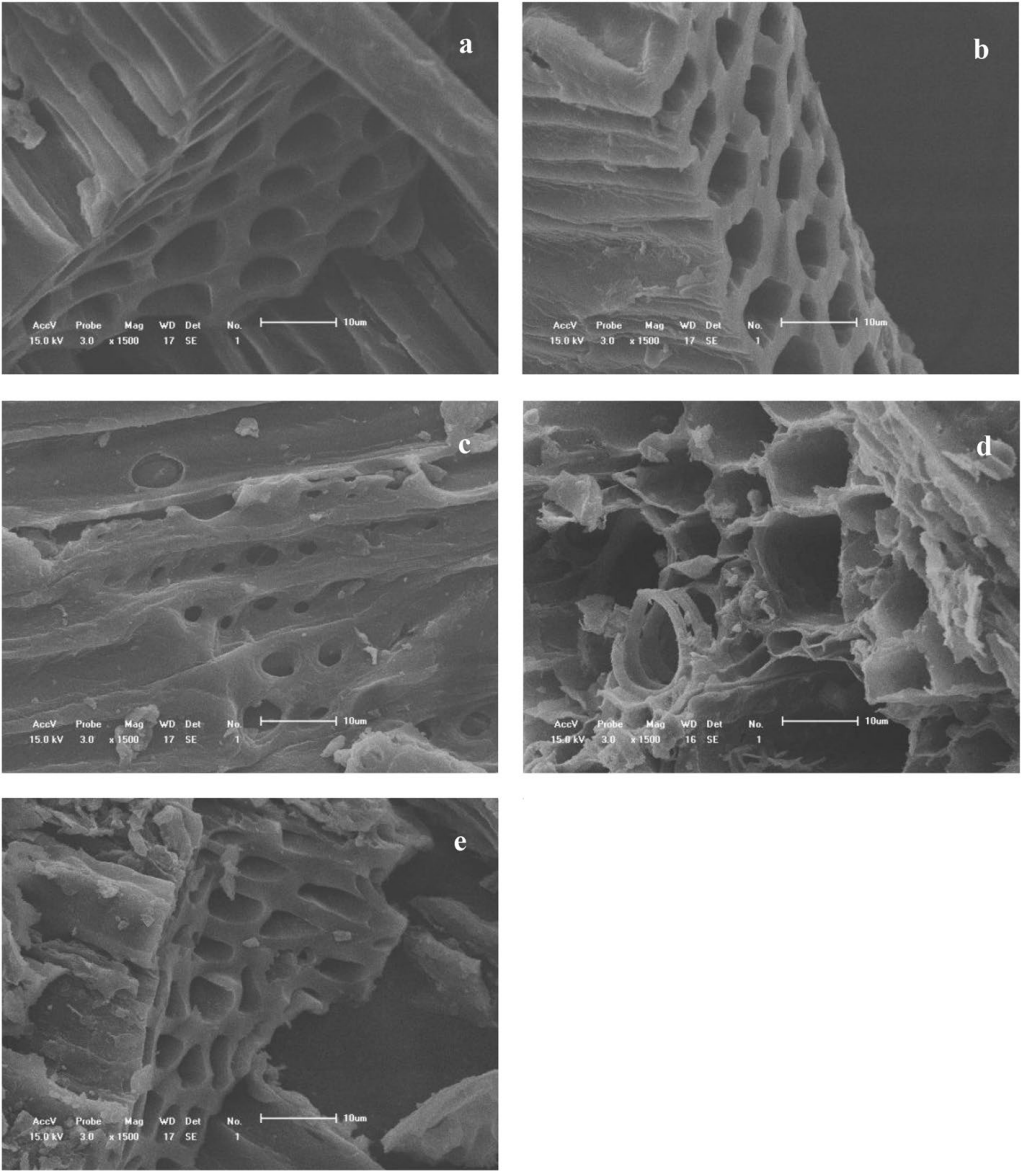
Electron micrographs of different straw biochar. Letter in the picture stands for: (**a**) corn stalk biochar (1500 × magnification); (**b**) Rice straw biochar (1500 × magnification); (**c**) cotton straw biochar (1500 × magnification); (**d**) Peanut shell biochar (1500 × amplification); (**e**) Wheat straw biochar (1500 × magnification).

Among the five carrier materials, corn straw biochar, rice biochar and wheat straw biochar showed the best properties. At 7 d, the water absorption rates of the three carrier materials were 86.3%, 64.1% and 58.6%, respectively (Table 2), and the antibacterial activities were 80.4%, 72.2% and 64.6%, respectively. When stored at room temperature (25 °C ± 5 °C), corn straw biochar had the highest effective number of living bacterial cells of all five types of carrier material. The pore layered structure of corn straw biochar, rice straw biochar and wheat straw biochar formed a complex three-dimensional structure, indicating that the three kinds of biochar had a highly porous structure (Fig. 2a,b,e). Such structure is expected to be beneficial to the adhesion and reproduction of bacteria, and the diffusion of primary and secondary metabolites supporting the normal metabolism of the introduced biocontrol strain. In contrast, cotton biochar (Fig. 2c) had relatively sparse pore structures, and peanut shell biochar (Fig. 2d) did not show pore structures suitable for the survival of microorganisms, suggesting bacteria could only attach to the surface of biochar, making for a poor carrier. Based on these results, biochars derived from corn, rice or wheat straw were selected as the most optimal carrier materials for producing microbial inocula.

**Table 2:** Adsorption stability of different carrier materials.

Stalk biochar	WA(%)	FIR(%)	Effective number of live cells (10 4 cfu/g)				
			1d	7d	14d	21d	28d
Corn	86.3 a	80.4 a	36.6 ± 0.3 a	85.1 ± 1.2 a	105.2 ± 1.5 a	113.8 ± 1.3 a	101.3 ± 1.1 a
Rice	64.1 b	72.2 b	35.5 ± 0.8 b	54.9 ± 0.6 c	76.1 ± 0.9 c	88.7 ± 1.0 b	85.8 ± 0.8 b
Cotton	35.9 d	43.1 d	9.2 ± 0.6 d	21.7 ± 0.6 d	26.3 ± 0.5 d	19.2 ± 0.8 d	18.4 ± 0.5 d
peanut shell	17.7 e	39.7 e	8.6 ± 0.5 e	7.4 ± 0.5 e	6.1 ± 0.3 e	5.6 ± 0.5 e	4.8 ± 0.2 e
Wheat straw	58.6 c	64.6 c	22.4 ± 0.6 c	63.5 ± 0.8 b	87.7 ± 1.2 b	82.2 ± 0.4 c	79.5 ± 0.3 c

*WA* water absorption, *FIR* fungal inhibition rate.

### Effects of microbial inocula on physicochemical properties and enzyme activities of ginseng soil

At 28 days, microbial inocula significantly altered the characteristics of the soil (Table 3). Compared with the control group, the treatments with wheat straw biochar, rice straw biochar and corn straw biochar increased the pH by 4.43%, 6.55% and 11.18%, and organic matter by 7.43%, 22.10% and 55.43%, respectively. At the same time, there were significant differences among the four treatments. The contents of total nitrogen, available nitrogen, available phosphorus, and available potassium in the soil in the corn straw biochar treatment were significantly increased by 33.07%, 26.70%, 16.40%, and 9.10%, respectively, compared with the control group. Microbial inocula had significant effects on the activities of urease, sucrase and catalase in the soil (Table 3), but had no significant effect on the activity of phosphatase. Compared with the control group, the urease, catalase and sucrase activities in the soil in the corn straw biochar treatment were increased significantly (by 52.73%, 16.80% and 43.80%, respectively).

**Table 3:** Effect of biological control agents on repairing the diseased soil.

Treatment	A	B	C	CK
pH value	5.42 ± 0.05 c	5.53 ± 0.03 b	5.77 ± 0.08 a	5.19 ± 0.06 d
OM (g kg-1)	17.21 ± 0.45 c	19.56 ± 0.47 b	24.90 ± 0.55 a	16.02 ± 0.60 d
TN (g·kg-1)	1.42 ± 0.06 b	1.33 ± 0.08 c	1.69 ± 0.06 a	1.27 ± 0.05 d
AN (mg kg-1)	136.33 ± 9.05 c	132.95 ± 7.48 b	156.20 ± 8.36 a	123.28 ± 7.95 d
AP (mg kg-1)	24.38 ± 0.46 b	22.18 ± 0.35 c	25.98 ± 0.41 a	22.32 ± 0.52 c
AK (mg kg-1)	160.65 ± 4.83 c	163.87 ± 6.32 b	172.90 ± 5.69 a	158.48 ± 6.56 d
URE (mg/g·d-1)	25.78 ± 0.49 b	24.12 ± 0.54 bc	30.21 ± 0.56 a	19.78 ± 0.75 d
CAT (g/mL)	1.63 ± 0.04 b	1.57 ± 0.06 c	1.73 ± 0.03 a	1.48 ± 0.05 d
INV (mg/g·d-1)	18.12 ± 0.63 b	16.42 ± 0.66 c	22.03 ± 0.45 a	15.32 ± 0.37 d
NPH (mg/g·d-1)	0.42 ± 0.03 ab	0.41 ± 0.02 ab	0.45 ± 0.03 a	0.39 ± 0.04 b

A treatment is wheat straw biochar preparation of microbial inocula; B treatment is preparation of microbial inocula by rice straw biochar; C treatment is corn stalk biochar preparation of microbial inocula; CK treatment is do not add any substance as blank control.

*OM* organic matter, *TN* total nitrogen, *AN* available nitrogen, *AP* available phosphorus, *AK* available potassium, *URE* urease activity, *CAT* catalase activity, *INV* invertase activity, *NPH* neutral phosphatase activity.

### Composition of soil bacterial community

**Figure 3:**
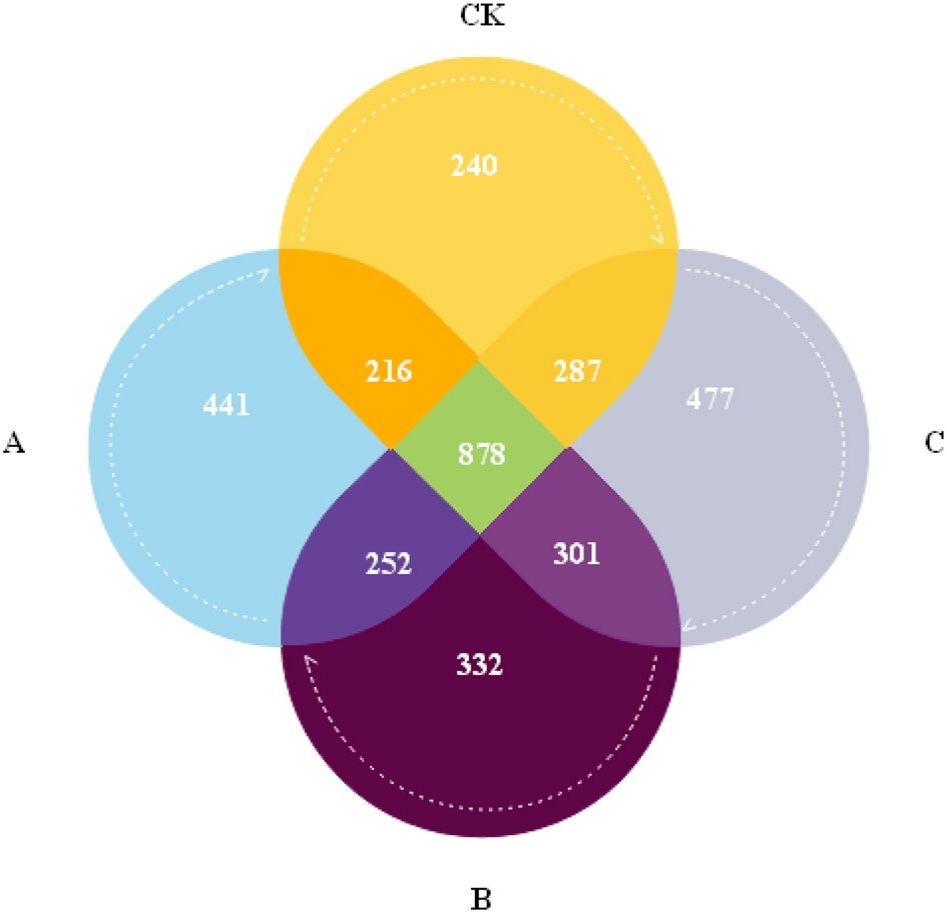
OTUs number of bacterial communities in each treatment. Using BMGE1.12 software (https://bioweb.pasteur.fr/packages/pack@BMGE@1.12) to create.

**Figure 4:**
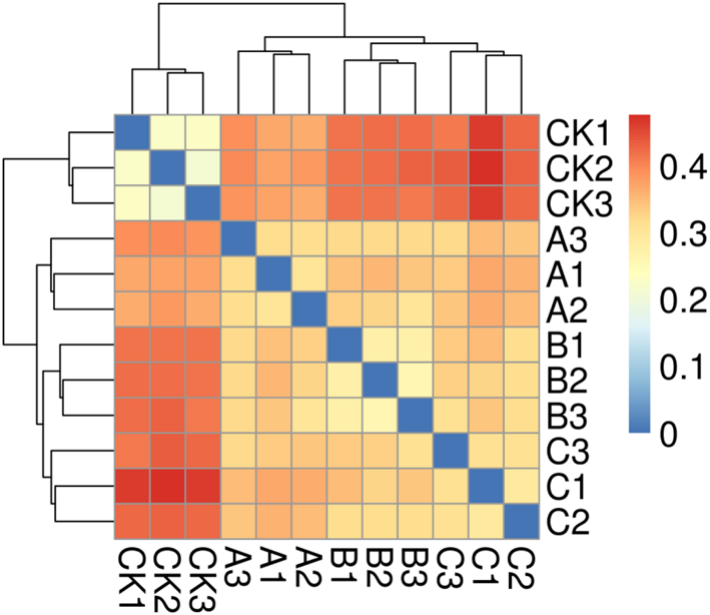
Heatmap of each treatment of ginseng soil. Bray Curtis algorithm was used to calculate the distance between the two samples and obtain the distance matrix. The distance among samples is represented by a color gradient. Created using Mothur1.43.0 software (http://mothur.org).

The four soil treatments showed 421,879 effective bacterial sequences and 7,114 OTUs (Fig. 3). Compared with the unamended control group, total OTUs in the wheat straw biochar, rice straw biochar and corn straw biochar treatments increased by 10.24%, 8.76% and 19.86%, respectively (Table 4). Shannon and Simpson indices reflect the diversity of taxa, and these indices were significantly increased in comparison to those in the control group (P < 0.05). In order to characterize the differences between treatments, we used the Bray–Curtis test to quantify the sample distances (Fig. 4). The results indicated that the differences among different treatments were significant. Compared with the control treatment (CK), the distances were large in the treatments with wheat straw biochar and corn straw biochar and small with the rice straw biochar treatment.

**Table 4:** Effect of different treatments on the α diversity of bacterial community.

Sample	Number	Chao1	Coverage	OTUs	PD-whole-tree	Shannon	Simpson
A	148,035 ± 1138	1842.39 ± 98.11	0.987	1787 ± 73 a	94 ± 6	8.89 ± 0.43 b	0.98
B	134,853 ± 1281 a	1732.94 ± 112.15c	0.987	1763 ± 101c	81 ± 4 cd	7.68 ± 0.37 c	0.96
C	15,309 ± 1023 cd	1987.33 ± 102.32 a	0.988	1943 ± 89 a	103 ± 5 a	9.43 ± 0.35 a	0.98
CK	123,682 ± 1302 b	1712.43 ± 92.37 bc	0.985	1621 ± 79 c	77 ± 5 c	7.32 ± 0.48 d	0.95

### Clustering analysis of bacterial community at the class level

**Figure 5:**
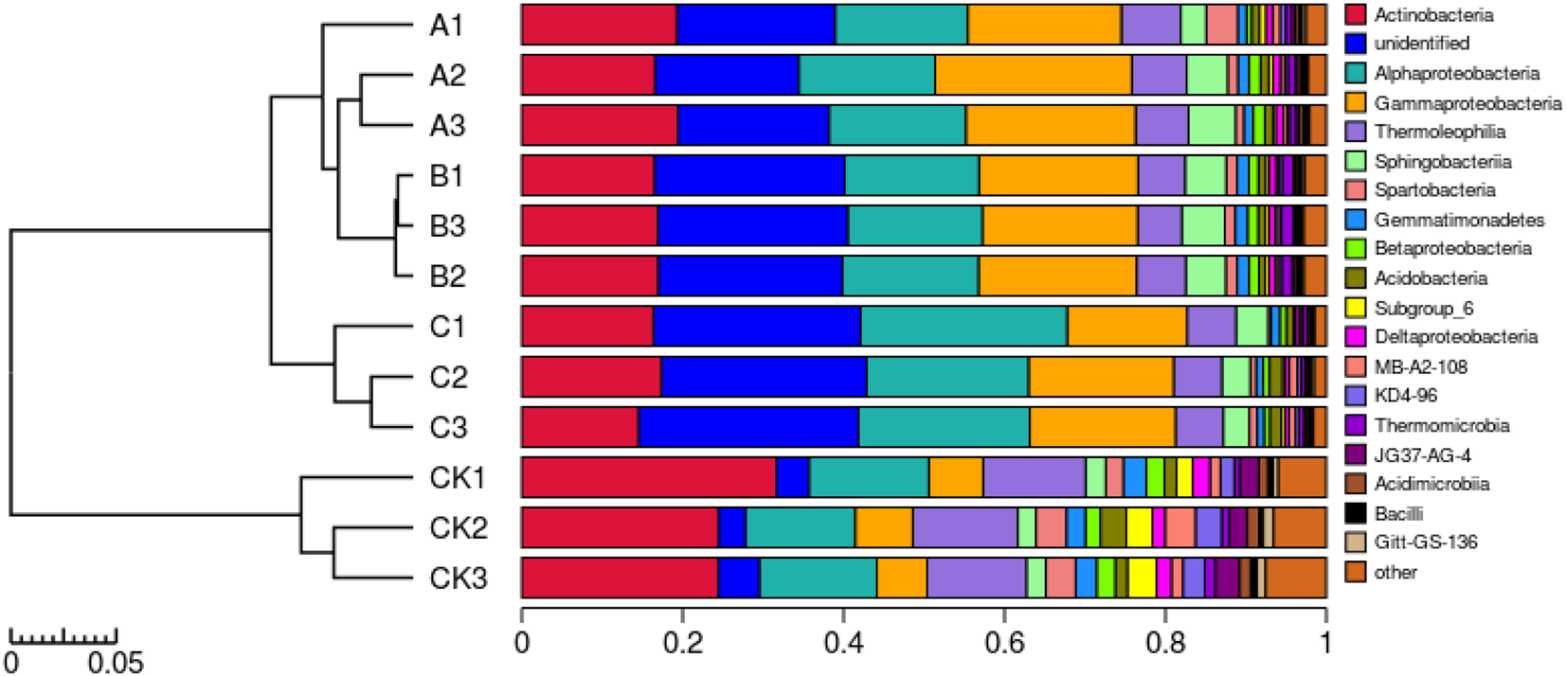
Analysis of relative abundance of bacterial community in ginseng soil at the class. Created using R3.6.0 software (https://www.r-project.org).

In all the soil samples, we detected 39 phyla, 97 classes, 153 orders, 225 families, and 306 genera. The twelve soil samples from four different treatments were divided into two categories (Fig. 5). The soil samples from the unamended control were clustered into one branch, whereas the soil samples from the wheat straw biochar, rice straw biochar and corn straw biochar treatments were clustered into another branch. The relative abundance of bacterial community composition was analyzed at the class level, and there were five dominant phyla (abundance of > 2%) whose relative abundance in the soil was significantly different among the treatments. Compared with the control, the relative abundance of *Alphaproteobacteria, Gammaproteobacteria and Sphingobacteria* was significantly higher, and the relative abundance of *Actinobacteria* and *Thermoleophilia* was significantly lower. In three of the treatments, the relative abundance of class *Actinobacteria* accounted for 18.29% in the wheat straw biochar treatment, almost 16.53% in the rice straw biochar treatment and 15.62% in the corn straw biochar treatment. Compared with the control, the corn straw biochar treatment increased the abundance of *Alphaproteobacteria, Gammaproteobacteria* and *Sphingobacteria* by 7.87%, 9.81% and 1.24%, respectively. In summary, the effects of the treatments wheat straw biochar and rice straw biochar on the improvement of bacterial community were similar, and both of them mainly enhanced the relative abundance of *Alphaproteobacteria* and *Gammaproteobacteria*.

**Figure 6:**
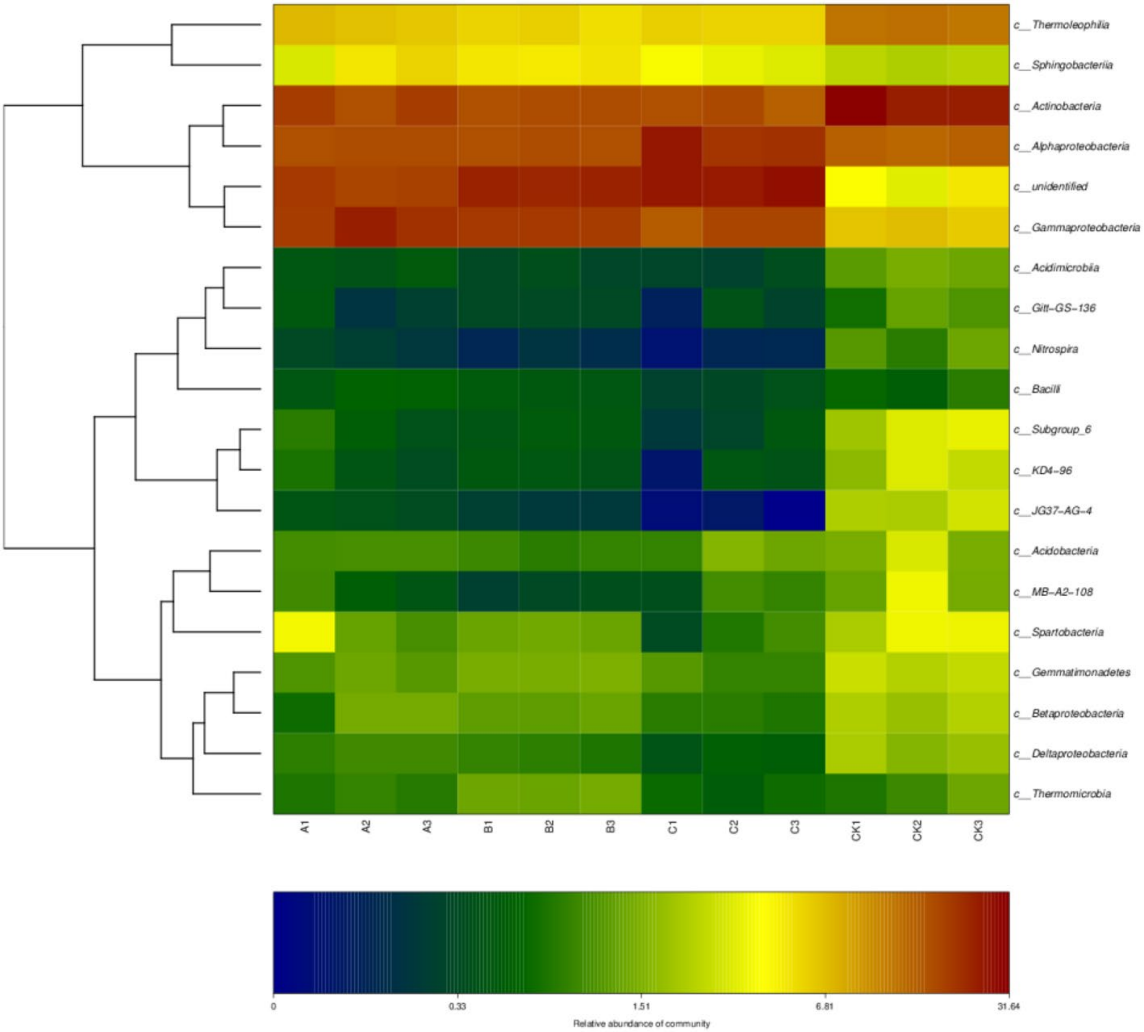
Abundance thermogram and cluster map of the top 20 samples at the class level. Created using Mothur1.43.0 software (http://mothur.org). Here, only the heatmap of OTUs of TOP20 and their gate levels are shown. The horizontal axis represents samples at different points, the vertical axis represents OTUs of different points, and the depth of color represents the abundance.

The soil samples from different treatments were clustered by taxa, or similarity of abundance among samples, and the clustered data were used to construct a hierarchical clustering heatmap (Fig. 6). The soil bacterial communities treated by wheat straw biochar, rice straw biochar and corn straw biochar were grouped into two groups, one of which was the community with high relative abundance of *Thermoleophilia, Sphingobacteria, Actinobacteria, Alphaproteobacteria*, and *Gammaproteobacteria*.

### Significant differences in taxa among the treatment groups

**Figure 7:**
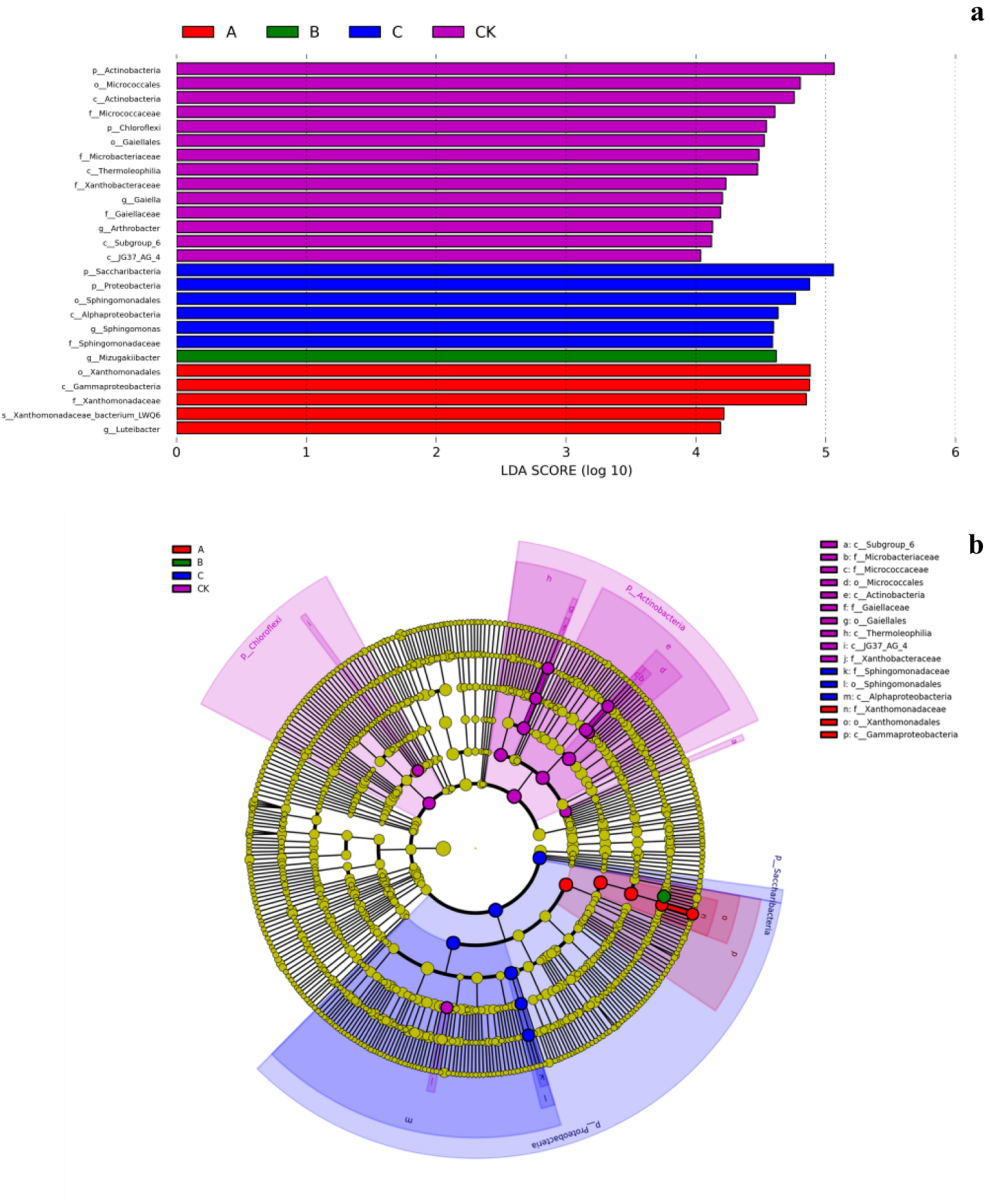
Classification level discriminant analysis (LDA) (**a**) and class level LEfSe analysis (**b**). Using LEfSe1.1.0 software (http://huttenhower.sph.harvard.edu/lefse) to create. (**a**) shows the biomarker with statistical difference for the taxa with LDA score greater than the set value. The default value is 4.0 (see the abscissa, only the absolute values of LDA greater than 4 are shown in the figure). The color of the bar chart represents each group, and the length of the bar chart represents the size of the LDA score, which represents the impact size of the taxa with significant differences. (**b**) the circles radiating from the inside to the outside represent taxonomic levels from phylum to genus (or taxa). Each small circle at different taxonomic levels represents a taxon at that level, and the diameter of the small circle is positively correlated with the relative abundance. Color: taxa with no significant difference are uniformly colored in yellow, and the biomarker taxa are colored according to the group. The red nodes represent the taxa with significant differences in the red group, and the green nodes represent the taxa with significant differences in the green group.

Linear discriminant analysis (LDA), which allows comparisons among the treatment groups, also performs subgroup comparisons within the group comparisons to find taxa with significant differences in abundance among the groups. When LDA > 4.0, there were 26 taxa groups with significant differences among wheat straw biochar, rice straw biochar, corn straw biochar, and CK at each classification level (Fig. 7a); the CK had the most significant differences with 14 taxa. Among the other three treatments, five taxa were significantly different in the wheat straw biochar treatment, and the rice straw biochar treatment had 1. There were many significantly different taxa in the corn straw biochar treatment; at LDA > 4.0 it had six and at LDA > 5.0 it had one *Saccharibacteria*. These results indicated that *Saccharibacteria* contributed greatly to the significance of differences and was the most important taxa that caused the differences among the four treatments.

LEfSe analysis showed (Fig. 7b) there were 24 significantly different taxa among the four treatments, including three in the wheat straw biochar treatment, whereas rice straw biochar had zero, corn straw biochar had 3, and CK had 18. The number and abundance of different taxa were the highest in the CK treatment. Different taxa treated in the wheat straw biochar treatment were mainly *Xanthomonadaceae, Xanthomonadales and Gammaproteobacteria*, and in the corn straw biochar treatment were mainly *Sphingomonadaceae, Sphingomonadales* and *Alphaproteobacteria*. After treatments wheat straw biochar, rice straw biochar, corn straw biochar, and CK, the bacterial taxa with the largest contribution were *Proteobacteria, Actinobacteria, Chloroflexi*, and *Saccharibacteria*, respectively.

### Redundancy analysis (RDA) of bacterial community structure and soil environmental factors

**Figure 8:**
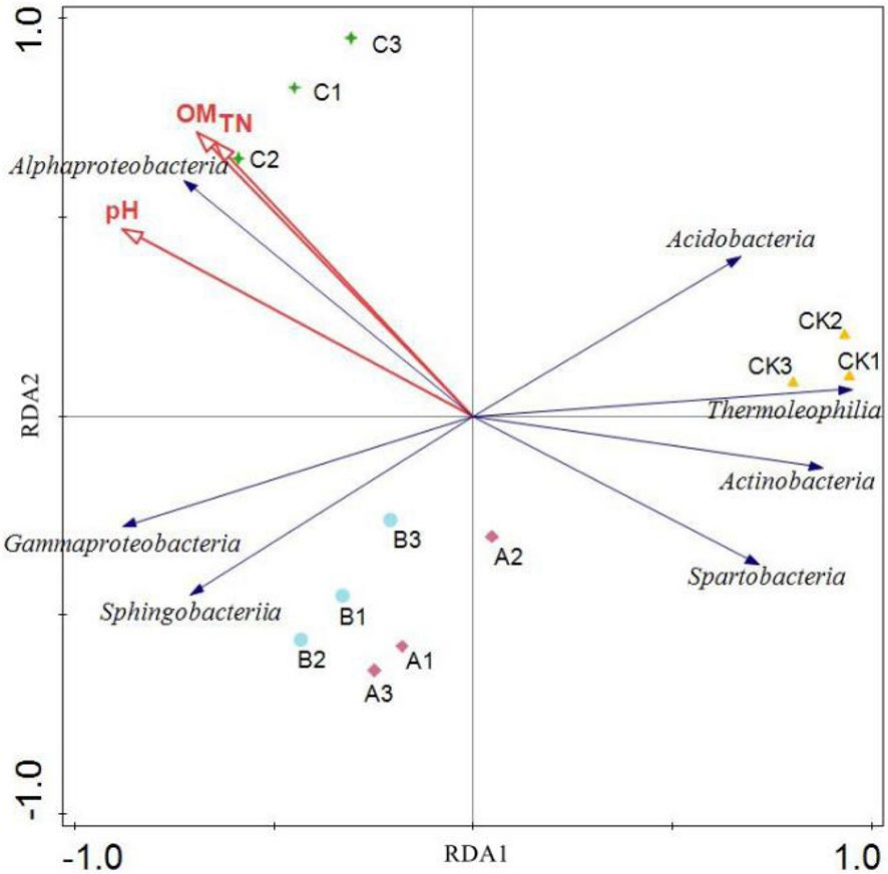
Redundancy analysis of soil bacteria community and soil physicochemical factors. Environmental factors including the pH value, soil organic matter (OM) and soil total nitrogen (TN).

Soil pH, organic matter (OM), total nitrogen (TN), and dominant taxa in the bacterial community were analyzed by RDA (Fig. 8). Organic matter and total nitrogen were the main factors influencing bacterial community composition in ginseng soil after different treatments. In the RDA analysis, the distance between the corn straw biochar treatment and CK was large, and the two treatments occupied separate quadrants, indicating that the soil bacterial community structure of the corn straw biochar and CK treatments was significantly different. The treatments with wheat straw biochar and rice straw biochar occupied a common quadrant, indicating the similar bacterial community structure. The correlation analysis showed that *Alphaproteobacteria* were significantly positively correlated with organic matter and total nitrogen (P < 0.05). *Acidobacteria, Thermoleophilia, Actinobacteria,* and *Spartobacteria* were negatively correlated with organic matter and total nitrogen (P < 0.05).

## Discussion

Reasons for the problems associated with continuous cropping are extremely complex, involving soil, plants, microorganisms, and many other aspects. Some studies have shown that the change of soil physical and chemical properties and the lack of soil fertility are the main reasons for problems with continuous cropping^31^. Microbial fertilizer is a living-bacteria preparation that may have many functions, such as enhancing substrate fertility, promoting nutrient absorption by plants, and improving disease resistance of crops^32^. In the present study, the overall remediation effect in soil produced by the microbial inoculum composed of *Frankia* F1 met the agricultural requirements. The *Frankia* F1 strain showed high inhibitory effects on *Fusarium solani*, *Sclerotinia schinseng*, *Cylindrocarpon destructans*, *Alternaria panax*, and *Rhizoctonia solani*, with inhibition rates of 80.23%, 73.91%, 72.12%, 70.87%, and 68.31%, respectively. Out of the five types of biochar tested, three types of high-quality biochar were selected as the carrier for microorganisms. Due to its porous nature, biochar can provide attachment sites and large space for the survival of microorganisms, and can be used as a good carrier for plant probiotics or other microorganisms^33,34^. In this study, the biochar prepared with corn straw, wheat straw and rice straw were applied to the soil; they improved soil pH value, organic matter, fertility, and enzyme activity. Yulan et al.^35^ found that, compared with organic fertilizer application alone, the mixed application of organic fertilizer and microbial inoculum could greatly improve the content of organic matter and available phosphorus in the soil of an abandoned greenhouse. Therefore, the application of microbial inocula can significantly improve the soil nutrient status and increase crop yield. This may be because the application of microbial inocula can significantly enhance soil enzyme activity and promote the transformation of nutrients into available forms^36^.

To probe the microbial diversity more deeply, we conducted 16S rRNA analysis on the soils. In the ginseng soil tested in our study, the top five classes of bacteria included *Actinobacteria* (26.54%), *Alphaproteobacteria* (14.88%), *Gammaproteobacteria* (6.84%), *Thermoleophilia* (12.38%), and *Sphingobacteria* (2.44%). Different biochars with *Frankia* F1 strain had different effects on various microbial taxa. After the corn straw biochar treatment, the abundance of *Alphaproteobacteria, Gammaproteobacteria* and *Sphingobacteria* increased by 7.87%, 9.81% and 1.24%, respectively. *Alphaproteobacteria* and *Gammaproteobacteria* belong to *Proteobacteria*, and they include nitrogen-fixing bacteria that form symbiosis with plants^37^. *Gammaproteobacteria* often utilize nutrients such as ammonia and methane generated by the decomposition of organic substances^38,39^. *Sphingobacteria* are conducive to the degradation of cellulose in litter. *Actinobacteria* can promote the rapid decay of litter and are important participants in the decomposition of chitin and lignin^40^.

The four soil treatments generated 421,879 effective bacterial sequences and 7,114 OTUs (Fig. 3). The relative abundance Chao1 index and the diversity index of bacterial community in soil increased after wheat straw biochar, rice straw biochar and corn straw biochar treatments (Table 4). This is consistent with the research results of Guangming^41^, with the addition of biochar improving the population structure diversity of soil microorganisms in ash desert soil. The promotion effect of biochar on soil bacterial community may be attributed to the following reasons: biochar provides more carbon sources for soil bacteria and improves the environmental conditions for soil bacteria; the complex pore structure of biochar provides a good habitat for the growth of soil bacteria and protects them from being preyed upon by other organisms^42^. *Frankia* F1, which has antifungal effect, was selected as a biocontrol microorganism. It is an actinomycete that can become a symbiont with non-legumes to form root nodules and fix atmospheric nitrogen. It forms vesicles on the straw at the top of the mycelium. It has a very high nitrogen fixation efficiency and a wide range of host plants across different families. Studies on the influence of biochar on soil bacteria mostly focus on nitrogen bacteria. Many scholars have found that nitrogen bacteria can promote nitrogen fixation and inhibit denitrification^43^.

In ecology, redundancy analysis is a method to identify the relationship between environment, taxa and vegetation. The bacterial taxa in soils were closely related to the pH value, the total carbon, and the total nitrogen in the soils^44^. Using the redundancy analysis, Jian et al.^45^ concluded that soil organic carbon and total nitrogen were soil indicators in the process of rocky desertification in mountainous karst areas. In this study, redundancy analysis was conducted on bacterial community structure and the soil environmental factors. The RDA results showed that pH value, organic matter and total nitrogen were the main environmental factors affecting the community composition of ginseng soil bacteria. Studies have shown that soil pH, organic carbon and nitrogen are important environmental factors that affect the composition of bacterial communities^46^, and organic matter can increase the activity of soil enzymes and improve soil biological activity^47^ by regulating the capacity of soil microorganisms to utilize carbon sources^48^. Fertilization can not only change soil physical and chemical properties, but also regulate soil enzyme activities and promote soil microorganisms coordinating nutrient cycling in soil.

Biochar is relatively stable, which significantly affects the activities of soil microorganisms. The pore structure of biochar and its adsorption of water and fertilizer can provide a good habitat for microorganisms^49^. Jin et al.^50^ found that the addition of biochar could significantly increase soil microbial biomass nitrogen proportionally with an increase in the amount of biochar. The application of biochar can cause changes in the bacterial community structure in the soil, and the bacterial community structure is significantly correlated with the soil pH value and nutrients^19,51^.

## Conclusion

Use of corn straw biochar and *Frankia* F1 to prepare a composite microbial inoculum is very important for restoration of ginseng soil chemical and biological environment. In this study, corn straw biochar showed better porosity and biocompatibility than rice straw biochar, cotton biochar, peanut shell biochar, and wheat straw biochar, making corn straw biochar suitable for the adhesion and survival of *Frankia* F1. Compared with the other four kinds of biochar, the corn straw biochar used to prepare a composite microbial inoculum with *Frankia* F1 had the best load and highest fungal inhibition rate. In addition, soil chemical properties and soil enzyme activity showed the optimum after 28 d of microbial inoculum treatment, gradually enriching the soil microbial community and improving its structure in ginseng soil, and increasing the relative abundance of beneficial bacteria. It is concluded that the proportion of biocontrol bacteria and beneficial bacteria in soil can be controlled by adjusting soil pH value and organic matter and total nitrogen contents in the production practice. In summary, the changes in the bacterial compositions in our study were caused by the application of the microbial inoculum prepared with microorganisms and biochar. Therefore, the application of corn straw biochar and *Frankia* F1 has an application potential in efficiently repairing the chemical and biological environment of ginseng soil. This study provides a theoretical basis for the development and application of ginseng microbial inoculum.

## Methods

### Soil collection and preparation of related materials

Soil samples were collected in Jilin Province, China (126° 44′ 22′′ E and 42° 39′ 51′′ N). The samples were obtained randomly by dividing a 100 m^2^ field of continuous ginseng cropping into 10 small blocks. In each block, 5 soil samples (> 1 kg) were randomly taken from 0 to 20 cm depth. The physicochemical properties of ginseng soil were pH value 4.79, organic matter 14.67 g/kg, total nitrogen 0.81 g/kg, available nitrogen 122.43 mg/kg, available phosphorus 21.93 mg/kg, and available potassium 143.82 mg/kg. The chemicals used in this study were all analytical grade. The Frankia F1 strain was provided by the Department of Environmental Engineering, College of Resources and Environment, Jilin Agricultural University, China. We utilized NCBI-BLAST for the highly homologous gene sequences in the database, and used the MEGA 7.0 software to construct the phylogenetic tree. Ginseng pathogens *Fusarium solani*, *Cylindrocarpon destructans*, *Phytophthora cactorum*, *Alternaria panax*, *Rhizoctonia solani*, *Sclerotinia schinseng,* and *Botrytis cinerea* were provided by Plant Pathology Laboratory of College of Agronomy, Jilin Agricultural University, and stored at 4 °C on agar slants at low temperature.

### Media

The culture media were as follows:

Gao's No. I solid medium: soluble starch 20.0 g, KNO_3_ 1.0 g, NaCl 0.5 g, K_2_HPO_4_ 1.0 g, MgSO_4_ 0.5 g, FeSO_4_ 0.01 g, pH = 7.0, agar 20.0 g, distilled water 1000 mL.

Gao's No. I fluid medium: Gao's No. I solid medium without agar.

Potato dextrose agar (PDA) medium: 200 mL potato juice, 20 g glucose, 20 g agar, pH = 7.0. All media were autoclaved at 121 °C for 20 min before use.

Verification of Frankia F1 against pathogenic fungi of ginseng.

Filter paper method^52^ was used to make bacterial plates of each activated pathogen. Four sterilized filter paper circles with a diameter of 1 cm were placed to the four points 25 mm away from the center of the plate. The filter papers were infused with 20 μL *Frankia* F1 bacterial suspension and the control treatment with 20 μL sterile water. Cultures were incubated at 28 °C for 7 days. The presence of fungistatic bands was observed, and the diameter of pathogenic bacteria colonies was measured to calculate the fungistatic rate.

### Preparation of microbial inocula

First, *Frankia* F1 was inoculated in 100 mL of Gao's No. I fluid medium and incubated at 30 °C for 7 days. Corn straw biochar, rice straw biochar, peanut shell biochar, wheat straw biochar, and cotton biochar were selected as candidate materials for the preparation of microbial inocula. The hydroscopicity of the carrier material, the biocompatibility with *Frankia* F1 and the fungistatic activity of the prepared microbial inoculum against pathogenic fungi were determined, and the surface morphology of the candidate material was visualized using a scanning electron microscope (JSM-7800F, Japan).

Next, each of the 5 carrier materials was sterilized in a 50 mL centrifuge tube with 2 g of the material, and then mixed with 10 mL *Frankia* F1 bacterial suspension (1 × 10^9^ CFU/mL), put in a sterile bottle, dried in oven at low temperature (30 °C), and stored at 4 °C and 25 ± 5 °C (room temperature). All five types of microbial inocula were prepared in the same way.

Finally, at days 1, 7, 14, 21, and 28, the number of living cells in different types of carrier materials was determined by the plate counting method, and the antibacterial activity was determined by the plate-disk incubation method at 7 d.

### Remediation of ginseng soil with microbial inocula

The pot experiment was carried out in a solar greenhouse of Jilin Agricultural University College of Resources, Environment and Environment in September 2020. The whole experiment lasted 28 days. The objective of this study was to evaluate the application of different types of straw-derived biochar (corn straw biochar, rice straw biochar, peanut shell biochar, wheat straw biochar, and cotton biochar) in combination with antagonistic *Frankia* F1. The effects of microbial inoculum on physicochemical properties, fertility, enzyme activities, and bacterial community structure of continuous cropping ginseng soil were studied. Four treatments were set, featuring microbial inocula prepared with different biochars derived from wheat (treatment A), rice (treatment B), corn straw (treatment C), and unamended control (CK).

The soil (1 kg per pot) was mixed with a treatment inoculum (10 g) and placed into a pot. Each treatment had five independent repetitions, and a total of 20 planting pots were set up. The soil in each of the planting pot was watered to about 75% water holding capacity by adding distilled water every day by weighing. At the end of the experiment, 3 replicates from each treatment were randomly selected for subsequent analyzes. The samples were equally divided into two parts: one part was frozen at − 80 °C for DNA extraction and another part was preserved at 4 °C for further analysis. The physicochemical properties and enzyme activities of soil were measured by the published methods^53–55^.

### DNA extraction and PCR amplification

Soil samples were collected from four different treatments, and DNA was extracted from the soil using a PowerSoil DNA separation kit (MoBio Laboratories, Carlsbad, CA, USA) in accordance with the protocol recommended in the instructions. The purity and quality of genomic DNA were determined by 1% agarose gel. Target fragments in the v3-v4 regions of the bacterial 16S rRNA hypervariable region were amplified with universal primers 338F(5'-ACTCCTACGGGAGGCAGCA-3') and 806R(5'-GGACTACHVGGGTWTCTAAT-3'). A 10-digit barcode sequence was added to the 5 'end of the positive and negative primers of each soil sample (provided by Owesen, Beijing, China). The PCR amplification was performed by a Mastercycler (Eppendorf, Germany), and the amplification procedure was pre-denaturation at 95 °C for 5 min. There were 32 cycles at 95 °C for 45 s, 55 °C for 50 s and 72 °C for 45 s followed by elongation at 72 °C for 10 min. Each sample was repeated 3 times. After amplification, PCR products from the same sample were mixed. The PCR products were detected by electrophoresis, and the target band sizes were amplified by 1% agarose gel electrophoresis, and purified by an Agencourt AMPure XP nucleic acid purification kit and sent to Orvison, Beijing, China, for Illumina Miseq high-throughput sequencing.

### Statistical analysis

The raw data were first screened and the sequences were removed if they were shorter than 200 bp, had a low quality score (≤ 20), contained ambiguous bases, or did not exactly match the primer sequences and barcode tags. Qualified reads were separated using the sample-specific barcode sequences and trimmed with Illumina Analysis Pipeline Version 2.6. Then the dataset was analyzed using vsearch. The sequences were clustered into operational taxonomic units (OTUs) at a similarity level of 97% followed by generating rarefaction curves and calculating the abundance and diversity indices. The Ribosomal Database Project Classifier tool was used to classify all sequences into different taxonomic groups^56^. To examine the similarity between different samples, the clustering analyses and PCA were conducted based on the OTU information from each sample using R^57^. The evolution distances between microbial communities from each sample were calculated using the tayc coefficient and were represented by the Unweighted Pair Group Method with an Arithmetic Mean (UPGMA) clustering tree describing the dissimilarity among multiple samples^58^. To compare the membership and structure of communities in different samples, heat maps were generated with the top 20 OTUs using Mothur^59^. The LEFSe component of Galaxy software was used to analyze the significant differences in soil bacterial community composition and abundance in different treatments^60^.

## References

[1] Briskin DP (2000). Medicinal plants and phytomedicines. Linking plant biochemistry and physiology to human health. Plant Physiol..

[2] Shibata S (2001). Chemistry and cancer preventing activities of ginseng saponins and some related triterpenoid compounds. J. Korean Med. Sci..

[3] Yuan HD, Kim JT, Kim SH (2012). Ginseng and diabetes: The evidences from in vitro, animal and human studies. J. Ginseng Res..

[4] Li C, Yan Z, Zhang L (2014). Research and implementation of good agricultural practice for traditional Chinese medicinal materials in Jilin Province, China. J. Ginseng Res..

[5] Liu M, Li S, Xing Y, Ma F (1984). Identification of ginseng rust Rot fungus. J. Plant Pathol..

[6] Liu Z, Chen X, Han Y (2017). Research on Ginseng rust rot pathogen under natural overwintering conditions. Northern Horticult..

[7] Wang Q, Sun H, Xu C (2019). Analysis of rhizosphere bacterial and fungal communities associated with rusty root disease of Panax ginseng. Appl. Soil. Ecol..

[8] Rafael L-C, Juan Arturo R-S, Montserrat C-S (2020). Microencapsulation of Meyerozyma guilliermondii by spray drying using sodium alginate and soy protein isolate as wall materials: A biocontrol formulation for anthracnose disease of mango. Biocontrol Sci. Technol..

[9] Moparthi S, Bradshaw M (2020). Fungicide efficacy trials for the control of powdery mildew (*Podosphaera cerasi*) on sweet cherry trees (*Prunus avium*). Biocontrol Sci. Tech..

[10] Zhou CY, Xu SQ, Yan MX, Cui LL, Hua LL, Wang YP (2020). Identification and optimization of fermentation conditions of antagonistic endophytic fungi in a single plant of Panax ginseng. Henan Agricult. Sci..

[11] Sun Z, Yang LM, Han M, Han ZM, Yang L, Cheng L, Yang X, Lv ZL (2019). Biological control ginseng grey mold and plant colonization by antagonistic bacteria isolated from rhizospheric soil of Panax ginseng Meyer. Biol. Control.

[12] Kambo HS, Dutta A (2015). A comparative review of biochar and hydrochar in terms of production, physico-chemical properties and applications. Renew. Sustain. Energy Rev..

[13] Lehmann J, Gaunt J, Rondon M (2006). Bio-char sequestration in terrestrial ecosystems—a review. Mitig. Adapt. Strat. Glob. Change.

[14] Uzoma KC, Inoue M, Andry H (2011). Effect of cow manure biochar on maize productivity under sandy soil condition. Soil Use Manag..

[15] Baiamonte G, Pasquale CD, Marsala V (2015). Structure alteration of a sandy-clay soil by biochar amendments. J. Soils Sedim..

[16] Lehmann J (2007). Bio-energy in the black. Front. Ecol. Environ..

[17] Ding Y, Liu J, Wang Y (2013). Effects of biochar on soil microbial ecology. Chin. J. Appl. Ecol..

[18] Solaiman ZM, Blackwell P, Abbott LK (2010). Direct and residual effect of biochar application on mycorrhizal root colonisation, growth and nutrition of wheat. Soil Res..

[19] Zheng J, Chen J, Pan G (2016). Biochar decreased microbial metabolic quotient and shifted community composition four years after a single incorporation in a slightly acid rice paddy from southwest China. Sci. Total Environ..

[20] Gul S, Whalen JK, Thomas BW (2015). Physico-chemical properties and microbial responses in biochar-amended soils: Mechanisms and future directions. Agr. Ecosyst. Environ..

[21] Zhang W, Chen W, Meng J, Jin L, Guo W, Zhao H (2019). Utilization potential, industrial model and development strategy of straw biochar in Northeast China. Sci. Agric. Sin..

[22] Mao H (2018). Improvement of biochar and bacterial powder addition on gaseous emission and bacterial community in pig manure compost. Bioresour. Technol..

[23] Yumin D (2019). Positive impact of biochar alone and combined with bacterial consortium amendment on improvement of bacterial community during cow manure composting. Bioresour. Technol..

[24] Ghodhbane-Gtari F, Beauchemin N, Bruce D (2013). Draft Genome Sequence of *Frankia* sp. Strain CN3, an Atypical, Noninfective (Nod-) Ineffective (Fix-) Isolate from Coriaria nepalensis. Genome Announc..

[25] Jung-Tai L, Sung-Ming T (2018). The nitrogen-fixing *Frankia* significantly increases growth, uprooting resistance and root tensile strength of Alnus formosana. Afr. J. Biotech..

[26] Du D, Yuan F, Li R, Wang Y, Cui G (1985). A study on the classification and identification of a *Frankia* strain. Acta Microbiol. Sin..

[27] Kang L, Li S, Peng Y, Liu Y, Chen H, Luo C (2000). Field study on inoculation of Casuarina casuarina with Franklinella calcium alginate. Forest Res..

[28] Larkin RP (2003). Characterization of soil microbial communi- ties under different potato cropping systems by microbial population dynamics, substrate utilization, and fatty acid profiles. Soil Biol. Biochem..

[29] Shi L, Du N, Shu S (2017). Paenibacillus polymyxa NSY50 suppresses Fusarium wilt in cucumbers by regulating the rhizospheric microbial community. Sci. Rep..

[30] Shen Z, Ruan Y, Chao X (2015). Rhizosphere microbial community manipulated by 2 years of consecutive biofertilizer application associated with banana Fusarium wilt disease suppression. Biol. Fertil. Soils.

[31] Atandi JG, Haukeland S, Kariuki GM, Coyne DL, Karanja EN, Musyoka MW, Fiaboe KKM, Bautze D, Adamtey N (2017). Organic farming provides improved management of plant parasitic nematodes in maize and bean cropping systems. Agricult. Ecosyst. Environ..

[32] Wang T, Qiao W, Li Y, Ao Y (2011). Effects of crop rotation and microbial fertilizer on soil physical and chemical properties and biological activity of cucumber continuous cropping. Chin. J. Soil Sci..

[33] Daquan SJ (2015). Effect of volatile organic compounds absorbed to fresh biochar on survival of *Bacillus mucilaginosus* and structure of soil microbial communities. J. Soils Sedim..

[34] Warnock DD, Lehmann J, Kuyper TW (2007). Mycorrhizal responses to biochar in soil—concepts and mechanisms. Plant Soil.

[35] Luo Y, Tian G, Zhang D, Hao R, Wang C (2015). Effects of microbial agents on soil nutrients and nitrate nitrogen accumulation in terracotta greenhouse. Chin. Agric. Sci. Bull..

[36] Yin S, Zhang L, Zhang G, Huang Y, Cui G, Liang R, Duan P, Cheng H, Ma Y (2012). Effects of complex ecological microbial agents on the number and enzyme activity of cucumber rhizosphere soil. Chin. J. Microbiol..

[37] Dedysh SN, Ricke P, Liesack W (2004). NifH and NifD phylogenies: An evolutionary basis for understanding nitrogen fixation capabilities of methanotrophic bacteria. Microbiology.

[38] Michael, P. C., Madigan, T., Martinko, J. M. & Parker, J. Getting the bug for microorganisms. In *Brock biology of microorganisms*, 8th edn. 375–376 10.1016/s0962-8924(97)83479-4 (Prentice Hall, 1997).

[39] Lv X, Yu J, Fu Y (2014). A meta-analysis of the bacterial and archaeal diversity observed in wetland soils. Sci. World J..

[40] Zhao Y, Wu F, Yang W (2015). Variations in bacterial communities during foliar litter decomposition in the winter and growing seasons in an alpine forest of the eastern Tibetan Plateau. Can. J. Microbiol..

[41] Jin Xu, Wang R, Deng F, Cao G, Wang G (2020). Effects of biochar application on soil physical and chemical properties and enzyme activities of poplar plantation in Dongtai coastal area. J. Fujian Agric. For. Univ..

[42] Lehmann J, Rillig MC, Thies J (2011). Biochar effects on soil biota—A review. Soil Biol. Biochem..

[43] Nugroho SG, Lumbanraja J, Suprapto H (1996). Three-year measurement of methane emission from an Indonesian paddy field. Plant Soil.

[44] Sauze J, Jérme O, Maron PA (2017). The interaction of soil phototrophs and fungi with pH and their impact on soil CO2, CO18O and OCS exchange. Soil Biol. Biochem..

[45] Long J, Liao H, Li J, Chen C (2012). Research on the relationship between soil and rocky desertification in typical karst mountain area based on redundancy analysis. Environ. Sci..

[46] Zeng J, Lou K, Zhang CJ (2016). Primary succession of nitrogen cycling microbial communities along the deglaciated forelands of tianshan mountain, China. Front. Microbiol..

[47] Sui Y, Jiao X, Gao C, Cheng W, Zhang X, Liu X (2009). Study on the relationship between soil organic matter content and soil microbial biomass and soil enzyme activity. Chin. J. Soil Sci..

[48] Jiao X, Gao C, Sui Y, Zhang X, Ding G (2011). Sci. Agric. Sin..

[49] Yao L, Cheng G, Wang L, Chen H, Lou L (2015). Effects of biochar application on soil microorganisms. Environ. Chem..

[50] Rui J, Kexing L, Shaoying Ai, Linfeng Li, Mingming T, Yanhong W, Chao Li, Jianfeng N (2016). Effects of biochar on soil properties, cadmium uptake and physiological characteristics of Chinese cabbage. J. Southern Agric..

[51] Zheng H, Honghui Wu, Wengi B, Ye J, Zeng Y (2019). Soil Fertil. Sci..

[52] Shan W, Li J, Liu M (2010). Inhibition of Verticillium wilt in cotton by filter paper method. Chin. Agric. Sci. Bull..

[53] Kızılkaya R, Aşkın T, Bayraklı B, Sağlam M (2004). Microbiological characteristics of soils contaminated with heavy metals. Eur. J. Soil Biol..

[54] Lee SH, Lee S, Kim DY (2007). Degradation characteristics of waste lubricants under different nutrient conditions. J. Hazard. Mater..

[55] Zhang YM (2005). Changes in enzyme activities of spruce (Picea balfouriana) forest soil as related to burning in the eastern Qinghai-Tibetan Plateau. Appl. Soil. Ecol..

[56] Cole JR, Wang Q, Cardenas E, Fish J, Chai B, Farris RJ, Kulam-Syed-Mohideen AS, McGarrell DM, Marsh T, Garrity GM (2009). The ribosomal database project: Improved alignments and new tools for rRNA analysis. Nucleic Acids Res..

[57] Wang Y, Sheng HF, He Y (2012). Comparison of the levels of bacterial diversity in freshwater, intertidal wetland, and marine sediments by using millions of illumina tags. Appl. Environ. Microbiol..

[58] Jiang XT, Peng X, Deng GH (2013). Illumina sequencing of 16S rRNA tag revealed spatial variations of bacterial communities in a mangrove wetland. Microb. Ecol..

[59] Jami E, Israel A, Kotser A (2013). Exploring the bovine rumen bacterial community from birth to adulthood. ISME J..

[60] Nicola S, Jacques I, Levi W (2011). Metagenomic biomarker discovery and explanation. Genome Biol..

